# Modulation of oxidative stress and microinflammatory status by colloids in refractory dialytic hypotension

**DOI:** 10.1186/1471-2369-12-58

**Published:** 2011-10-20

**Authors:** Guy Rostoker, Mireille Griuncelli, Christelle Loridon, Thomas Bourlet, Eric Illouz, Abbes Benmaadi

**Affiliations:** 1 Service de Néphrologie et de Dialyse, Hôpital Privé Claude Galien, 20 route de Boussy, 91480 Quincy sous Sénart, France; 2 Service de Cardiologie, 20 route de Boussy Saint Antoine, 91480 Quincy sous Sénart, France

**Keywords:** dialytic hypotension, 4% gelatin, 20% hyperoncotic albumin, microinflammatory status, oxidative stress

## Abstract

**Background:**

Intradialytic hypotension may adversely affect the outcome of chronic hemodialysis. Therapeutic albumin has powerful anti-oxidant and anti-inflammatory properties. We have recently shown that systematic colloid infusion during hemodialysis sessions improves hemodynamic parameters in most dialysis hypotension-prone patients unresponsive to usual of preventive measures.

We postulated that frequent hypotensive episodes may lead to a noxious inflammatory response mediated by oxidative stress induced by ischemia-reperfusion. The aim of this study was therefore to analyze the effect of 20% albumin and 4% gelatin infusions on oxidative stress and microinflammatory status in hypotension-prone patients unresponsive to usual preventive measures.

**Methods:**

Prospective cross-over study (lasting 20 weeks) of routine infusion of 200 ml of 20% albumin versus 200 ml of 4% gelatin in 10 patients with refractory intradialytic hypotension. We analyzed the effect of 20% albumin and 4% gelatin on microinflammatory status, oxidative stress, serum nitrite and nitrate levels by analysis of variance.

**Results:**

A significant decrease in serum ceruloplasmin and serum C3 was observed during the albumin period (p < 0.05, repeated measure ANOVA). A significant decrease in serum hydrogen peroxide was seen during albumin and gelatin administration (p < 0.01, repeated measure ANOVA) and a very large decrease in serum lipid peroxides was observed during the albumin period only (p < 0.01, Friedman test). Serum lactoferrin, serum proinflammatory cytokines and serum nitrite and nitrate levels remained stable during the different periods of this pilot trial.

**Conclusions:**

We conclude that the improvement in microinflammatory status observed during colloid infusion in hypotension-prone dialysis patients may be related to a decrease in ischemia-reperfusion of noble organs, together with a specific reduction in oxidative stress by albumin.

**Trial registration:**

ISRCTN 20957055

## Background

Intradialytic hypotension is the most common complication of hemodialysis, affecting up to 33% of patients. It interferes with patients' well being and prevents adequate dialysis and fluid removal [1-3]. Intradialytic hypotension has a negative impact on health-related quality of life and reduces patients' life expectancy, favoring underdialysis and increasing the risk of ischemia or infarction of noble organs such as the heart, brain and bowel [2-4].

Recent studies have shown that human albumin can be safely used in intensive care patients [5] and exerts powerful anti-oxidant properties [6]. It may also better restore volemia and arterial pressure than saline derivatives in dialytic hypotension-prone patients, especially those with cardiovascular disease [7,8]. We recently conducted a 20-week a single-blind prospective cross-over pilot trial of systematic infusion of 20% albumin as compared to 4% gelatin in 10 hypotension-prone patients on long-term bicarbonate hemodialysis and unresponsive to cool dialysate associated with sodium and ultrafiltration profiling. We observed a potential benefit of systematic colloid infusion, especially 20% albumin, in this subset of patients [9]. We postulated that frequent episodes of hypotension may induce a noxious inflammatory response mediated by oxidative stress induced by ischemia-reperfusion.

The aim of the present study was to analyze the effect of systematic infusion of 20% albumin and 4% gelatin on micro-inflammatory status, oxidative stress, serum nitrates and nitrites in these hypotension-prone patients unresponsive to usual preventive measures.

## Methods

### Patients and dialysis

Ten patients (6 women, 4 men) undergoing chronic intermittent bipuncture bicarbonate hemodialysis three times per week on a Cimino-Brescia fistula were included.

All the patients gave their written informed consent to the study. Their median age was 71.5 years (range: 45-91 years) and the median time spent on hemodialysis was 4 years (range: 1-9 years). The main characteristics of the patients are summarized in Table 1.

**Table 1:** Characteristics of the patients

n°	Age (year)	Gender	Duration of dialysis (years)	Cause of renal failure	Dialysis membrane	Modality of dialysis	Risks factors for dialysis hypotension
1	45	F	9	SLE	Polysufone (ARYLANE H4 - Hospal)	4 h × 3/Week	Long duration of dialysis (9 years), SLE still active treated by steroïds and ciclosporin - Diastolic dysfunction

2	91	F	2	Atherosclerotic nephropathy	Diacetate (DICEA130-Baxter)	3 h × 3/Week	Very old age, severe ischemic cardiopathy - Diastolic dysfunction

3	67	F	6	Interstitial nephrotoxic nephritis (Ciclosporin)	PMMA (BKF16-Toray)	4 h × 3/Week	Cardiac graft for ten years-Diastolic dysfunction

4	87	M	2	Atherosclerotic nephropathy	Diacetate (DICEA 150-Baxter)	3 h × 3/Week	Very old age, severe ischemic cardiopathy-Diastolic dysfunction

5	76	M	3	Diabetes melitus type II	Triacetate (TRICEA210-Baxter)	4 h × 3/Week	Old age, diabetes, pace-maker, panhypotuitarism - Diastolic dvsfunction

6	84	F	3	Renal and urologic tuberculosis	Diacetate (DICEA 130-Baxter)	3 h30 × 3/Week	Very old age, severe ischemic and valvular cardiopathy - Diastolic dysfunction

7	71	M	5	Atherosclerotic nephropathy	Chemically modified Acetate (SMC 170 - Bellco)	4 × 3 h/Week	Old age, severe ischemic cardiopathy-Diastolic dysfunction

8	72	M	1	Myeloma	Triacetate (TRICEA210-Baxter)	3 h30 × 3/Week	Old age, severe ischemic cardiopathy, arythmia secondary to atrial fibrillation - chemotherapy for myeloma - Diastolic dysfunction

9	70	F	7	Analgesic nephropathy	Diacetate (DICEA 130-Baxter)	4 h × 3/Week	Old age, long duration of dialysis (7 years), arythmia secondary to atrial fibrillation-Diastolic dysfunction

10	50	F	6	Autosomic dominant polycystic kidney disease	PAN (NEPHRAL 300-Hospal)	4 h × 3/Week	Binephrectomy - Diastolic dysfunction

All the patients experienced a hypotensive episode more than once a week, as defined by a systolic pressure (SBP) < 100 mmHg or a fall in systolic pressure > 30 mmHg associated with symptoms of hypotension. All the patients were at their optimal dry weight, as shown by normovolemia on echography of the inferior vena cava and echocardiography after a dialysis session or the following morning after an evening dialysis session [10,11]. Hemodialysis was performed using an Integra module (Hospal, Lyon, France), with ultrapure dialysate (bacterial content: < 0.1 CFU/ml and endotoxin content < 0.03 U/ml; dialysis water supply from Gambro, Colombes, France); the blood flow rate was 250-300 ml/min and the dialysate flow rate was 500 ml/min.

Dialysate temperature, dialysate composition (505 A from Fresenius, Fresnes, France) and the sodium and ultrafiltration profiles did not differ between the four treatment periods. Details on the patients' clinical conditions are provided in a recently published article [9].

### Study protocol

#### Trial registration

This trial received approval (n°02-017) from the ethics committee of Henri Mondor University Hospital and was registered with the French Agency for Drugs and Health Devices AFSSAPS (n° 021059) and with ISRCTN register (reference 20957055).

SRCTN-NAPN-20

#### Study design

**Figure 1:**
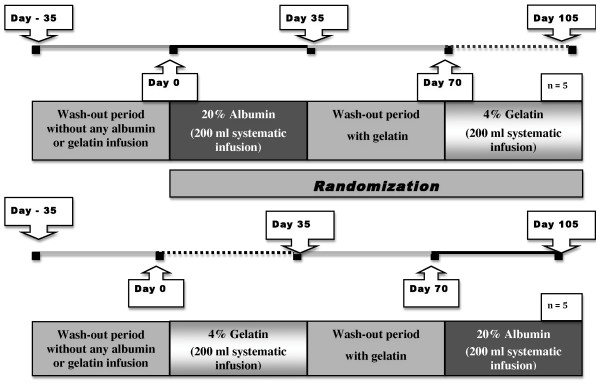
**Study Protocol**.

This was a randomized four-period cross-over study lasting 20 weeks. Patients were randomized to one of two treatment sequences (sequence 1 or sequence 2: Figure 1: see details of study protocol in reference 9).

Briefly, for sequence 1, after 35 days without treatment, the participants (n = 5) received an infusion of 200 ml of 20% albumin (Vialebex, LFB, Les Ulis, France) (from day 0 to day 35), followed by a washout period of 35 days, on 4% gelatin infusion (Gelofusin, Braun medical, Boulogne, France). During the last period from day 70 to day 105, the participants received systematic infusions of 200 ml of 4% gelatin.

For sequence 2, the participants (n = 5) received from day 0 to day 35 a systematic infusion of 200 ml 4% gelatin, followed by a washout period from day 35 to day 70 on 4% gelatin infusion; during the last period the participants received systematic infusions of 200 ml of 20% albumin.

Systematic infusion of 200 ml of 4% gelatin was required during the wash-out period by the ethics committee, to avoid noxious consequences of dialytic hypotension; the aim of this wash-out period was to avoid a carry-over effect of 20% albumin.

#### Outcome measures

The primary outcome measure was arterial pressure regulation and secondary outcome measures were nutritional status, ultrafiltration rate and dialysis quality, as recently reported [9]. Other secondary outcome measures, which included the effect of 20% albumin and 4% gelatin on microinflammatory status, oxidative stress, and serum nitrate and nitrite levels, are the subject of this study.

##### Study of microinflammatory status, oxidative stress, and serum nitrite and nitrate levels

Serum and plasma samples were prepared before and at the end of the last dialysis session in each of the four protocol periods. We analyzed micro-inflammatory status by measuring C-reactive protein, C3, haptoglobin and ceruloplasmin by nephelemetry (Minineph kits, The Binding Site Ltd; Saint-Egrève, France). We measured serum lactoferrin as a marker of polymorphonuclear neutrophil activation during dialysis sessions by using a home-made ELISA with rabbit anti-human lactoferrin and purified human lactoferrin purchased respectively from Biodesign International (Saco, USA) and Sigma-Aldrich (Saint Quentin Fallavier, France). We measured serum proinflammatory cytokines (IL1 beta, IL6, IL8, TNF alpha) as markers of monocyte activation during dialysis sessions, after blood collection in endotoxin-free tubes (Endo Tube ET, Chromogenix; Vienna, Austria), using ELISA kits (R and D systems; Lille, France). We analyzed oxidative stress by measuring circulating hydrogen peroxide and total lipid peroxides with colorimetric assays (Hydrogen Peroxide Kit, Assay Designs, Inc; Ann Arbor, USA and Per Ox Assay, Immunodiagnostik, Orange Medical, Brussels, Belgium). We measured nitrotyrosine, a stable endproduct of peroxynitrite oxidation, as a marker of NO-dependent damage in vivo, by using an ELISA method (HyCult biotechnology, Tebu-bio; Le Perray en Yvelines, France). We also analyzed the total antioxidant power of plasma after uric acid depletion (using rasburicase, 2.5 *μ*L/100 *μ*L of serum for one hour at 30°C: Fasturtec 1.5 mg/ml, Sanofi-Synthelabo, Paris France) by using a colorimetric assay based on the evaluation of Cu^+ ^derived from Cu^++ ^by the combined action of all antioxidants in the sample (Oxford Biomedical Research, Oxford, UK).

As NO has been implicated in the pathophysiology of dialytic hypotension, we measured serum nitrite and nitrate levels by spectrophotometry, based on the Griess reaction after extensive centrifugation of the samples at 4°C on Biomax-PB Ultrafree-MC centrifugal filter units with ultrafiltration membranes with a cut-off of 10 000 NMWL (Millipore, Saint-Quentin en Yvelines, France) and enzymatic conversion of nitrate to nitrite by nitrate reductase (Nitric oxide (NO2^-^/NO3^-^) assay (R and D systems; Lille, France).

##### Statistical analyses

We analyzed the effect of albumin and gelatin infusions on arterial pressure regulation (primary outcome measure), i.e. SBP (systolic blood pressure) and DBP (diastolic blood pressure), and the number of hypotensive episodes (defined as SBP < 100 mmHg, regardless of symptoms) and changes in the ultrafiltration rate and interdialytic weight gain (post-hoc analyses) in each individual (in both regular and additional dialysis sessions) by the n-of-1 trial methodology implemented with the Wilcoxon test. We also analyzed the effect of the colloids on dialysis quality (single pool Kt/V, ionic dialysance 30 minutes before the end of the dialysis session, decrease in relative blood volume) and nutritional parameters (serum albumin and prealbumin) by analysis of variance (see details in reference 9).

Changes in microinflammatory status, oxidative stress, serum cytokine, lactoferrin, nitrate and nitrite levels during the four periods of the protocol were analyzed with either parametric or non parametric analysis of variance followed by post-tests depending on the normality of the distribution (in the Kolmogorow-Smirnov test) [12] using Prism 4 software (Graphpad, San Diego, USA). P values < 0.05 were considered significant [12]. Values are expressed as means ± SD or medians and ranges, depending on the normality of the distribution [12].

## Results

### Hemodynamics, dialysis quality and nutritional parameters

Statistical analysis of individual data by the n-of-1 methodology showed that 20% albumin increased systolic blood pressure (SBP) in 6 patients (p < 0.05, Wilcoxon test) whereas 4% gelatin improved SBP in only 3 patients (p < 0.05, Wilcoxon test). Albumin infusions increased diastolic blood pressure (DBP) in 4 patients (p < 0.05, Wilcoxon test), whereas gelatin improved DBP in only 1 patient (p < 0.05, Wilcoxon test). Weight gain between dialysis sessions was generally similar during the periods in most patients. An increase in the ultrafiltration rate was observed in 5 of the 6 patients whose blood pressure was improved by colloids (p < 0.005, Wilcoxon test) lessening the need for additional dialysis sessions and reducing the difference between the target dry weight and weight measured at the end of the dialysis sessions. Kt/V and the fall in relative blood volume remained stable during the study, whereas ionic dialysance at the end of the dialysis sessions was improved only by albumin infusion (p < 0.05, repeated measures ANOVA). These results are extensively analyzed in reference 9.

### Microinflammatory status

**Figure 2:**
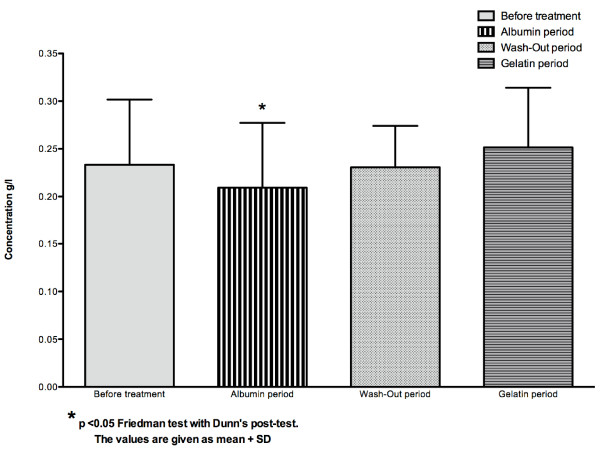
**Concentration of serum ceruloplasmin (g/l) in the whole group (n = 10)**.

C-reactive protein and haptoglobin levels did not differ between the four periods (p > 0.05 repeated measures ANOVA)(Table 2), whereas serum C3 and ceruloplasmin levels were significantly reduced during the albumin period (p < 0.05 non parametric ANOVA, Friedman test followed by Dunn's post test)(Figure 2).

**Table 2:** Inflammatory parameters during albumin and gelatin infusions

Parameter	Before treatment	Albumin infusion period	Wah-Out period	Gelatin infusion period	Statistical analyses
CRP(mg/l)	7.12 ± 8.01	9.25 ± 11.02	6.46 ± 7.10	4.78 ± 4.70	p > 0.05 at the repeated measures ANOVA

Ceruloplasmin (g/l)	0.22 [0.14 - 0.34]	0.19 [0.13-0.34]	0.23 [0.16 - 0.32]	0.23 [0.16-0.33]	p < 0.05 at the Friedman test

C3 (g/l)	0.97 [0.72-1.59]	0.78 [0.61-1.29]	0.96 [0.71-1.40]	0.96 [0.69-1.39]	p < 0.05 at the Friedman test

Haptoglobin (g/l)	1.35 ± 0.41	1.25 ± 0.50	1.22 ± 0.43	1.31 ± 0.41	p > 0.05 at the repeated mesures ANOVA

### Oxidative stress

**Figure 3:**
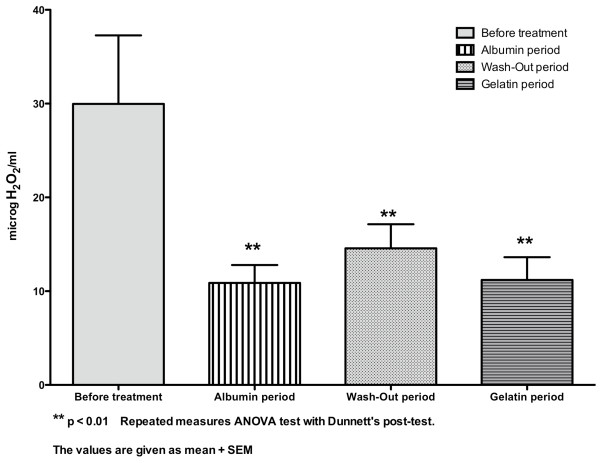
**Concentration of serum hydrogen peroxide (microH**_**2**_**O**_**2**_**/ml) in the whole group (n = 10)**.

**Figure 4:**
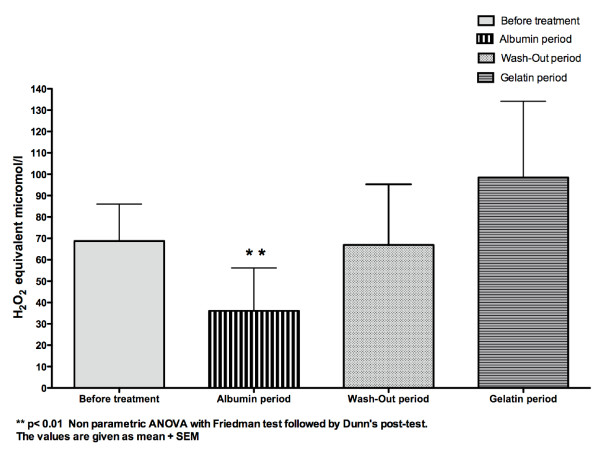
**Concentration of the serum lipid peroxides (H**_**2**_**O**_**2 **_**equivalent micromol/l) in the whole group (n = 10)**.

Plasma total antioxidant power did not differ between the four periods (p > 0.05 repeated measures ANOVA)(Table 3). Serum hydrogen peroxide values differed between the four periods (Table 3 and figure 3) (p < 0.01 repeated measures ANOVA) and were significantly lowered during the albumin period, wash-out period and gelatin period (p < 0.01 Dunnett's multiple comparisons test)(Figure 3). Serum lipid peroxides were strongly and significantly reduced only during the albumin period (p < 0.01 non parametric ANOVA Friedman test followed by Dunn's post-test) (Table 3 and figure 4). Nitrotyrosine was undetectable at the beginning of the dialysis sessions during the four periods (data not shown). Serum lactoferrin levels before and after dialysis sessions did not differ between the four periods (p > 0.05 repeated measures ANOVA)(Table 3).

**Table 3:** Oxidative stress, serum cytokines, serum nitrites and nitrates during albumin and gelatin infusions

Parameter	Before treatment	Albumin infusion period	Wah-Out period	Gelatin infusion period	Statistical analyses
Plasma total anti-oxydant power (copper reducing equivalent microM)	0.23 ± 0.08	0.26 ± 0.11	0.27 ± 0.13	0.22 ± 0.11	p > 0.05 at the repeated measures ANOVA

Hydrogen peroxyde μg/ml	29.97 ± 7.3	10.87 ± 1.9	14.57 ± 2.5	11.19 ± 2.4	p < 0.001 at the repeated measures ANOVA

Lipid peroxides (H202 equivalent) μM/1	50 [13.84-189.5]	7 [7-209.4]	37.30 [7-305]	38.20 [7.82-298.7]	p < 0.005 at the Friedman test

Lactoferrin at the beginning of the dialysis session (ng/ml)	245.7 ± 154	199.3 ± 133	154 ± 36.98	153.7 ± 29.79	p > 0.05 at the repeated measures ANOVA

Lactoferrin at the end of the dialysis session (ng/ml)	159.7 ± 40.52	144.5 ± 48	152.8 ± 31.42	154.9 ± 74.31	p > 0.05 at the repeated measures ANOVA

IL6 at the beginning of the dialysis session (pg/ml)	5.55 ± 8.19	2.44 ± 2.82	2.19 ± 2.87	1.74 ± 2.51	p > 0.05 at the repeated measures ANOVA

IL6 at the end of the dialysis session (pg/ml)	6.88 ± 7.56	6 ± 3.98	4.26 ± 2.19	5 ± 3.4	p > 0.05 at the repeated measures ANOVA

IL8 at the beginning of the dialysis session (pg/ml)	32.77 ± 43.79	21 ± 9.89	24 ± 18	19.83 ± 13.89	p > 0.05 at the Friedman test

TNF α at the beginning of the dialysis session pg/ml	9.85 ± 10.82	9.13 ± 5.87	8.04 ± 10.22	4.72 ± 3.59	p > 0.05 at the repeated measures ANOVA

TNF α at the end of the dialysis session pg/ml	8.33 ± 7.17	7.59 ± 4.67	5.40 ± 2.82	4.51 ± 2.96	p > 0.05 at the repeated measures ANOVA

Serum nitrates + nitrites at the beginning of the dialysis session (μmol/1)	49 ± 24.90	50.35 ± 30.87	53.66 ± 26	55.35 ± 28.42	p > 0.05 at the repeated measures ANOVA

Serum nitrates + nitrites at the end of the dialysis session (μmol/I)	45.34 ± 27.16	48.58 ± 17.78	47.27 ± 20	53.70 ± 34.48	p > 0.05 at the repeated measures ANOVA

The values are given either as mean ± SD in case of gaussian distribution or as medium [range] in case of non parametric distribution

### Serum cytokines

IL-1 beta was undetectable both at the beginning and end of the dialysis sessions during each period of the protocol (data not shown). Serum levels of the proinflammatory cytokines IL6, IL8 and TNF alpha did not differ between the four periods of the protocol at the beginning or end of the dialysis sessions (p > 0.05 repeated measures ANOVA)(Table 3).

### Serum nitrite and nitrate levels

The sum of serum nitrite and nitrate levels did not differ between the four periods of the protocol at the beginning or end of the dialysis sessions (p > 0.05 repeated measures ANOVA)(Table 3).

## Discussion

We recently showed in a pilot single-blind cross-over study, using n-of-1 trial methodology, that systematic infusion of 20% albumin or 4% gelatin during hemodialysis sessions in hypotension-prone patients unresponsive to preventive measures improves hemodynamic parameters (systolic and diastolic blood pressure), increases the ultrafiltration rate and reduces the number of hypotensive episodes [9]. N-of-1 trials, or individualized medication effectiveness tests, are the only way of resolving clinical uncertainty about whether expensive new treatments are truly effective for particular patients. Two provisos must be met: first, the health problem must be a chronic condition in which the outcome measure can be palliated but not removed; second, the effect of treatment should be quantifiable [13,14]. We used the n-of-1 methodology because, in a chronic illness such as renal failure requiring chronic hemodialysis, it can provide an objective basis for identifying treatment outcomes more subtle than death or survival for an individual patient with a rare condition such as refractory dialysis-related hypotension. Moreover, in the case of a new and expensive therapy such as 20% albumin (200 ml of 20% albumin costs 80 euros compared to 4 euros for 4% gelatin), n-of-1 trials can furnish powerful evidence for provision on an individual basis, allaying managerial and medical fears as to the cost of frequently ineffective therapies being applied to an expanding at-risk population [13,14].

In this complementary study, we now show that systematic infusions of 20% albumin and 4% gelatin in this subset of hemodialysis patients also improve the microinflammatory state and reduce the abnormal oxidative stress. Data on the association between inflammatory status and dialysis hypotension are scarce [15]. Tomita and coworkers compared nine patients with a history of intradialytic hypotension with eight patients without dialysis-associated hypotension and found a correlation between the levels of CRP and IL6 and the maximum percent change in mean arterial pressure over multiple dialysis sessions, suggesting that dialysis hypotension may trigger inflammation [15]. This is consistent with Bergamini et al, who found significant TNF-alpha release during hypotensive episodes [16].

In this pilot study, C3 and ceruloplasmin were significantly lowered during the albumin period but not during the gelatin period. This is consistent with recent studies using experimental models of hemorrhagic shock, which indicated that the type of resuscitation fluid greatly influences proinflammatory responses and especially neutrophil activation and nuclear factor-Kappa B gene transcription; albumin was found to be the least proinflammatory fluid [17,18]. Our patients' use of ultrapure dialysate may explain the low levels of CRP and proinflammatory cytokines (IL6, IL8 and TNF alpha), levels of which have been shown to be greatly influenced by the type of dialysate: indeed, standard dialysate containing endotoxin, but not ultrapure dialysate, has been shown to be a potent inducer of these mediators [19]. The systemic use of cool dialysate, together with the sodium and ultrafiltration profiles and colloid infusion may also have contributed to these low levels of CRP and pro-inflammatory cytokines [9].

Conversely, ex-vivo data suggest that uremia may increase vascular permeability [20], which may rise acutely during dialysis-associated hypotension via the release of mediators such as adenosine, aimed at preserving perfusion of noble organs [21]; in this setting, albumin may itself influence vascular integrity by binding to the interstitial matrix and subendothelium and by altering the permeability of these layers to large molecules and solutes; these effects may be mediated by the binding of arachidonic acid to albumin and by polynitroxylated albumin, which inhibits xanthine-oxidase-mediated adhesion of human neutrophils to endothelial cells [22].

We found that serum hydrogen peroxide levels were significantly lowered during both the albumin and the gelatin periods, suggesting that the improvement in hemodynamic parameters by colloids reduces oxidative stress related to the ischemia-reperfusion of noble organs that occurs during dialytic hypotension [23]. In addition to classical ischemia-reperfusion mechanism, by analogy with heart failure, entry of bacterial endotoxin during dialysis sessions might result from intermittent underperfusion of the intestine during dialysis-associated hypotension, leading to cardiac stunning and oxidative stress [24-26]. Thus, colloids may improve both systemic and intestinal perfusion and reduce gut ischemia [24-26]. These data also strongly suggest that dialytic hypotension may contribute in various ways to the overproduction of reactive oxygen species seen in end-stage renal failure patients, a multifactorial process mainly related to uremia itself, hemoincompatibility of the dialysis system and trace amounts of endotoxin in the dialysate [27].

In this pilot study, serum lipid peroxide levels were significantly reduced only during the albumin period. This is consistent with data showing that human serum albumin and bovine serum albumin provide protection from lipid peroxidation propagated by inorganic reactive oxygen species generated from xanthine oxidase/hypoxanthine in artificial systems [28] and that persistent hypoalbuminemia in hemodialysis patients is associated with peroxidation of erythrocyte membranes [29**]**. Moreover, albumin is the major extracellular source of reduced sulfhydryl groups (thiols), which are avid scavengers of reactive oxygen and nitrogen species; in this way albumin influences the redox balance [30,31].

The main limitations of this pilot study are its small size and the possibility that the difference in ultrafiltration rates between the study periods may have affected the validity of the results for both haemodynamics and inflammation.

## Conclusions

We have previously shown that systematic infusions of 20% albumin and 4% gelatin during hemodialysis sessions improve hemodynamic parameters and the ultrafiltration rate in most hypotension-prone dialysis patients unresponsive to usual preventive measures [9]. We observed a parallel improvement in microinflammatory status, which might be related to the decrease in both ischemia-reperfusion of noble organs and oxidative stress. Hyperoncotic 20% albumin was found to have more potent anti-inflammatory and anti-oxidative properties than 4% gelatin.

Further well-designed controlled trials with a sufficient number of patients are needed to confirm the efficacy of hyperoncotic 20% albumin and 4% gelatin in hypotension-prone dialysis patients.

## List of Abbrevations Used

ANOVA: Analysis of variance; CFU: Colony-Forming Unit; CRP: C-reactive protein; DBP: Diastolic Blood Pressure; ELISA: Enzyme-linked immunosorbent assay; IL1 beta: Interleukin 1 beta; IL6: Interleukin 6; IL8: Interleukin 8; Kt/V: Urea fractional clearance; NMWL: Nominal Molecular Weight Limit; NO: Nitric Oxide; SBP: Systolic Blood Pressure; SLE: Systemic Lupus Erythematosus; TNF alpha: Tumor Necrosis Factor alpha

## Competing interests

The authors declare that they have no competing interests.

## Authors' contributions

GR contributed to the conception and design of the study, statistical analysis, interpreting the data, reporting of the work and writing of the article. MG performed laboratory analysis of microinflammatory status and oxidative stress, statistical analysis, tables and figures. CL performed laboratory analysis of microinflammatory status and oxidative stress, statistical analysis, tables and figures. TB monitored the study in the dialysis center. EI contributed to the conception and design of the study and monitored the patients' hemodynamic status. AB contributed to the planning and conduct of the study. All authors read and approved the final manuscript.

## Pre-publication history

The pre-publication history for this paper can be accessed here:

http://www.biomedcentral.com/1471-2369/12/58/prepub
